# Dietary Supplementation with γ-Aminobutyric Acid Improves Growth, Digestive Enzyme Activity, Non-Specific Immunity and Disease Resistance against *Streptococcus iniae* in Juvenile Olive Flounder, *Paralichthys* *olivaceus*

**DOI:** 10.3390/ani12030248

**Published:** 2022-01-20

**Authors:** Nathaniel W. Farris, Ali Hamidoghli, Jinho Bae, Seonghun Won, Wonsuk Choi, Janka Biró, Seunghyung Lee, Sungchul C. Bai

**Affiliations:** 1 Feeds & Foods Nutrition Research Center, Pukyong National University, Busan 48547, Korea; nathanielwfarris@gmail.com (N.W.F.); alihamid@pukyong.ac.kr (A.H.); bjh2921@naver.com (J.B.); seonghun.won@cj.net (S.W.); thm622@naver.com (W.C.); 2 Research Centre for Aquaculture and Fisheries, Hungarian University of Agriculture and Life Sciences, 5540 Szarvas, Hungary; nagyne.biro.janka@uni-mate.hu; 3 Department of Marine Bio-Materials & Aquaculture, Pukyong National University, Busan 48513, Korea; 4 FAO World Fisheries University Pilot Program, Busan 48547, Korea

**Keywords:** GABA, microbiome metabolite, functional additives, *Streptococcus iniae*, flounder, lysozyme, amylase, non-specific immunity

## Abstract

**Simple Summary:**

γ-aminobutyric acid (GABA) is a very important biomolecule that is found in all lifeforms and serves innumerable essential biological functions in pathways ranging from neural transmission to metabolism and immunity. In recent years, GABA was identified as an important metabolite involved in the modulation of the gut microbiome, and even appetite, in fish. The current trial aims to assess the effects of GABA as a supplement for nutritionally important biomarkers of fish health. Our results show that approximately 229–282 mg/kg of the total dietary GABA has important benefits for juvenile olive flounder, most significantly with improved disease resistance against *Streptococcus iniae*.

**Abstract:**

Recent research is increasingly shedding light on the important role that microbial metabolites such as γ-aminobutyric acid (GABA) play in the context of nutrition, cognition, immune function, and the modulation of the gut microbiome. Yet, very few trials were conducted to assess the effects of its supplementation on biomarkers of fish health. Therefore, an eight-week feeding trial was devised to evaluate GABA supplementation in juvenile olive flounder, (*Paralichthys olivaceus*). A total of 630 fish with an average weight of 4.90 ± 0.10 g (±SD) were randomly assigned to one of seven triplicate groups and fed a non-GABA supplemented diet (CON, with 92 mg/kg GABA content), a positive control with 4 g/kg oxytetracycline (OTC), and five other diets supplemented with 50, 100, 150, 200 and 250 mg/kg GABA (corresponding to a total GABA content of 154, 229, 282, 327 and 352 mg/kg, respectively). Growth, blood chemistry, nonspecific immunity, digestive enzyme activity and disease resistance were assessed. The results showed that 100 and 150 mg/kg GABA supplementation consistently yielded significant improvements (*p* < 0.05) in growth, intestinal amylase, serum lysozyme, and survival against infection with *Streptococcus iniae*. Based on polynomial analysis, the optimal supplementation level was determined to be 237 mg/kg. These results support GABA as an important functional feed additive in juvenile olive flounder.

## 1. Introduction

Olive flounder, *Paralichthys olivaceus*, also known as Japanese flounder or bastard halibut, is a marine demersal, oceanodromous, large-toothed, left-eyed flounder species of high market value. It is native to the temperate/subtropical waters of the Western Pacific and can be found in waters ranging from as far north as the eastern coast of Russia, down to the warm waters of the South China Sea, with its highest population concentrated in the waters just off the southern tip of the Korean peninsula, Jeju Island, and the Japanese archipelago [1]. Even though this species represents a very important fishery for the capture industry, aquaculture has steadily superseded it as the primary source of production over the last 40 years [2]. As the olive flounder aquaculture industry grows, certain problems have become apparent, such as the need to improve feed formulations, reduce reliance on fishmeal, and bolster immune functionality to ward off disease. Unfortunately, the latter problem has resulted in the abuse of antibiotics such as oxytetracycline (OTC) in the industry, which is why many feeding trials with this species often contain OTC-supplemented diets as a positive control [3,4,5,6,7,8]. In an effort to reduce the use of antibiotics as well as improve growth and other health-related parameters, many trials were conducted that focused on a wide array of feed additives [1,7,9]. One feed additive that has received increasing interest is γ-Aminobutyric acid (GABA). GABA, also known as 3-Carboxypropylamine, 4-Aminobutanoic acid, or Piperidic acid, is a non-proteogenic amino acid (non-α amino acid). GABA is synthesized from glutamate via decarboxylation by glutamate decarboxylase (GAD) with vitamin B6 in the form of Pyridoxal 5′-phosphate (PLP) as a coenzyme [10]. Its appearance can be described as a beige-to-light-brown powder that is soluble in water and heat stable at temperatures of less than 80 °C for durations of less than 15 min [11].

Historically, much of the literature on GABA focused on its role in the regulation of the nervous system [12,13,14,15,16,17]. Research over the last seventy years found that GABA serves many important biochemical functions across all domains of life from single-cell organisms to human beings [18,19,20,21,22,23,24,25,26,27,28]. One of the most notable roles that GABA plays in vertebrates is the regulation of neuronal excitability and synaptic transmission by the inhibition of the action potential and its connection to the Tricarboxylic Acid Cycle (TCA) via “the GABA shunt” [29,30,31,32,33]. The inhibitory effects of GABA are balanced by glutamate, which serves as the principal excitatory neurotransmitter in what is known as the glutamine–glutamate/GABA cycle [34,35]. This makes GABA an important topic of study across all disciplines of biology, with promising applications, specifically in the field of nutrition. 

It has long been believed that the oral administration of GABA could produce anxiolytic effects in line with those of GABA synthesized in neural tissues [36] and even a reduction in systolic blood pressure [37]. Additionally, since GABA is a metabolite of bacterial metabolism in the gastrointestinal tract (GIT), there have been some important studies that evaluated its ability to influence digestive health in fish [28,38,39,40,41]. So far, there have only been a few studies that investigated the effects of GABA on the diets of teleost fish, yet the results are promising. For example, in a recent trial by Temu et al. [42], juvenile Nile tilapia, receiving a dose of between 144 and 197 mg/kg, experienced a significantly improved weight gain (WG) and improved superoxide dismutase (SOD) activity. In this trial, the optimal level was calculated as 158 mg/kg. In a following trial by Bae et al. [43], a diet containing a 158 mg/kg supplementation resulted in improved growth, enhanced intestinal villi length, trypsin activity, and disease resistance against *Edwardsiella tarda*. In a different trial by Wu et al. [44], juvenile grass carp, given a supplementation of 50–100 mg/kg, experienced significant increases in specific growth rate (SGR), hepatopancreatic SOD activity, and neuropeptide Y (NPY) and ghrelin mRNA expression in the brain. The optimal dietary level for juvenile carp in this trial was determined to be 87.5 mg/kg, according to quadratic regression analysis based on SGR. Additionally, the results of ghrelin mRNA expression found in this trial are also complemented in the study by Zhang et al. [45], which found that a supplementation of 84–89 mg/kg GABA increased feed intake in Chinese mitten crab (*Eriocheir sinensis*) by modulation of orexigenic, neural, signal-related genes. Furthermore, in a very recent trial by Li et al. [46], which explored GABA supplementation in juvenile turbot that were fed high soybean meal diets, it was found that 160 mg/kg of supplemental GABA resulted in significant decreases in pro-inflammatory cytokines, an increase in anti-inflammatory cytokines, and a reversal of microbiome dysbiosis resulting from a high-soybean-meal, experimental diet. In summary, these trials found that some major benefits of GABA supplementation were improved growth, antioxidant capacity, the modulation of the microbiome, and orexigenic/anorexigenic pathways. Though these trials are beginning to shed light on GABA’s benefits, the exact mechanism of this action is still very hard to determine due to GABA’s involvement in numerous biochemical pathways, varied concentrations in feed ingredients, and the limited knowledge of its effects on the GIT/microbiome of aquatic organisms. Some have hypothesized that this action could be, at least in part, due to its indirect effects on the central nervous system’s GABAergic signaling pathway, which the aforementioned trials seem to support [47,48]. 

Even though the number of studies in this area are limited among aquatic species, they continue to grow, and have attracted the attention of feed manufacturers as a functional feed additive due to its earlier success in terrestrial species [49,50,51,52]. Because of the recent interest in GABA as a feed additive for aquaculture species, there is a need to establish the optimal dietary supplementation levels of GABA for important species in the aquaculture industry across a broad range of parameters. The current trial was carried out to establish an optimal dietary level for GABA in a practical olive flounder diet as well as to determine its ability to bolster growth, digestive enzyme activity, immune response, and disease resistance. 

## 2. Materials and Methods

### 2.1. Diet Preparation and Experimental Design

Formulation of the experimental diets was based on previous studies at our institution to meet the nutritional requirements of juvenile olive flounder [1,43,53]. The γ-aminobutyric acid (GABA) was sourced from Milae Resources ML Co Ltd. (Seoul, Korea) and analyzed by high-performance liquid chromatography (HPLC), which established a purity of 76.5%. A basal diet without GABA was used as a negative control (CON); a positive control composed of CON + 4 g/kg oxytetracycline (OTC), and five other diets were prepared by adding 50, 100, 150, 200, and 250 mg/kg GABA at the expense of wheat flour (Table 1). All dry feed ingredients were combined and mixed using a planetary electric feed mixer (HYVM-1214, Hanyoung Food Machinery, Hanam, Korea), then fish oil was added slowly until completely homogenized throughout the feed mix. This mix was then moistened with water to approximately 25% of the dry feed weight. The moistened feed mix was then pelletized using a benchtop pelletizer (Baokyong Commercial Co., Busan, Korea) with a 2mm die. This produced uniform strands of feed which were broken into smaller pieces, then spread out on paper sheets in a room equipped with a dehumidifier. Once the feed was dried, it was individually bagged, labeled and stored at −20 °C.

Diets were analyzed at the Feeds and Foods Nutrition Research Center (FFNRC) to determine the proximate composition of crude protein, moisture, lipid, and ash (Table 1). Proximate composition analysis of the experimental diets was performed using standard methods [54]. The diet samples were dried to a constant weight at 105 °C to determine their moisture content. Ash was determined by incineration at 550 °C with a muffle furnace (DAIHAN, WiseTherm^®^, Seoul, Korea). The content of crude lipid was analyzed by the Soxhlet extraction method utilizing the Soxtec system 1046. The content of crude protein was determined by the Kjeldahl method (N × 6.25) following acid digestion (Table 1). This method was also used to determine the proximate composition of the whole-body of the experimental fish. 

An additional sample of each diet was sent to the National Instrumentation Center for Environmental Management, College of Agriculture and Life Sciences at Seoul National University (Seoul 151–742, Korea), where the total levels of dietary GABA in the feed were determined to account for naturally occurring concentrations in the diet (Table 1) via high-performance liquid chromatography (HPLC). 

### 2.2. Experimental Fish and Feeding Trial

The eight-week feeding trial was conducted at the Dept. of Marine Bio Materials and Aquaculture, Pukyong National University (PKNU), Busan, Korea. Initially, over 2000 juvenile olive flounder, averaging 2.0 g in weight were purchased from SamBu farm (Chungcheong province, Korea) and brought to PKNU where they were carefully stocked into several 250L tanks. Fish were allowed to acclimatize to experimental conditions for 3 weeks, and fed a commercial diet to bring them up to the desired average initial weight of approximately 5.0g in weight, at which time they were fasted for one day prior to stocking. All fish used in this trial were clinically healthy and absent of lesions. A total of 630 fish with an average weight of 4.90 ± 0.10 g were divided into 21 groups of 30 individuals, stocked into 40 L (0.153 m^2^) tanks with a flow rate of 2 L/min and supplied with air stones. The stocking density was an average of 147.13 g/tank at 0.962 kg/m^2^ approximating guidelines established by Bai et al. [53]. This resulted in a starting percent coverage area (PCA) of 38%, as determined by analysis of the average total body coverage area of the fish during stocking. Water temperature was maintained at approximately 20 ± 1 °C [55]. Fish were slowly hand-fed the experimental diets to apparent satiation. The experimental diets were consumed readily by all fish, and researchers reported no apparent differences in feeding behavior among tanks. This is important when feeding juvenile olive flounder, since the pelletized feed descends in the water column, and due to their feeding behavior, multiple individuals often target the same feed as they dart upwards from the bottom of the tank to intercept it. Thus, care was taken to ensure feed was equally distributed across the area of the tank at a rate appropriate for the fish to consume. Any feces and uneaten feed was removed by siphoning immediately after the feeding period. The amounts of uneaten feed were negligible. As a summary, no apparent differences in feed intake were observed among treatment groups, and the average daily feed consumed for the duration of the trial was roughly 3.2% biomass, falling in line with the National Institute of Fisheries Science (NIFS; [56]) feeding guidelines.

### 2.3. Sample Collection and Analysis

Upon the conclusion of the eight-week feeding trial, fish were fasted for 24 h, then moved to shallow flat-bottomed basins of water from their respective tanks. The final weight and number of individuals in each tank were recorded for calculation of the final weight (FW), weight gain (WG), specific growth rate (SGR), feed efficiency (FE), protein efficiency ratio (PER), and survival. In addition, fish were placed in clear plastic basins with standard 2 × 2 cm graph paper laminated to the bottom. A photograph was taken to be used for analysis of the percent coverage area (PCA) in each respective tank as an additional measure of stocking density. PCA = (total area of flounder blindside in cm^2^/total area of tank bottom in cm^2^) × 100. All other indices were calculated by using the following equations [57]:
WG (%) = (final wt. − initial wt.) × 100/initial wt.
SGR (%/day) = (ln (final wt.) − ln (initial wt.)) × 100/days of feeding
FE (%) = (final wt. − initial wt.) × 100/dry feed intake
PER = (final wt. − initial wt.)/protein intake
Survival (%) = (total fish − dead fish) × 100/survival fish


Three fish from each tank were selected at random, anesthetized with 2-phenoxyethanol (200 mg/L for 5–10 min), individually weighed, and then the liver and intestines were removed for determination of the hepatosomatic index (HSI) and visceral somatic index (VSI). Blood samples were also taken using a non-heparinized needle from each fish, pooled according to tank, and allowed to coagulate at room temperature for 30 min. Blood serum samples were then centrifuged at 5000× *g* for 10 min and immediately stored at −70 °C for further analysis of non-specific, immune, antioxidant capacity, and blood-chemistry-related parameters, including lysozyme, myeloperoxidase (MPO), superoxide dismutase (SOD), alanine aminotransferase (ALT), aspartate aminotransferase (AST), total protein (TP) and glucose (GLU), respectively. Three specimens were kept from each tank for determination of whole-body proximate composition.

For digestive enzyme activity analysis, three fish were randomly collected from each tank (n = 3), anesthetized; then, intestines were removed and added to an assay buffer provided by each respective enzyme activity colorimetric assay kit (amylase and lipase) in proportions prescribed by the manufacturer (BioVision Incorporated, Milpitas, CA, USA). Samples were homogenized and centrifuged for 10 min. The supernatant was then transferred to 1.5 mL microcentrifuge tubes and a colorimetric assay was performed according to the manufacturer’s instructions. 

Lysozyme activity of the serum was analyzed by the use of a turbidimetric assay employing the methods described by Hultmark [58] with slight modifications. Briefly, *Micrococcus lysodeikticus* (0.75 mg/mL) was suspended in a sodium phosphate (PO_4_^3−^) buffer (0.1 M, pH 6.4). Next, 200 μL of this resulting suspension was aliquoted into each well of a 96-well microplate. Lastly, 20 μL of serum was added to the wells, then read at a wavelength of 570 nm after incubation at room temperature (25 °C), both initially (0 min) and then after 30 min had passed, using a microplate reader (UVM 340, Biochrom, Cambridge, UK). A reduction in absorbance of 0.001 min^−1^ is regarded as one unit of lysozyme activity.

MPO activities of serum were measured according to Quade et al. [59]. That is, 20 μL of serum was diluted with Hanks Balanced Salt Solution (HBSS), absent of Ca^2+^ or Mg^2+^ (Sigma-Aldrich, Burlington, MA, USA), in a 96-well microplate. Next, 35 μL of 3,3′,5,5′-tetramethylbenzidine hydrochloride (TMB, 20 mM) (Sigma-Aldrich, Burlington, MA, USA) and H_2_O_2_ (5 mM) were added. After 2 min had passed, the color change reaction was halted by the addition of 35 μL of 4 M sulfuric acid. The optical density was read at a wavelength of 450 nm. 

SOD activity of serum was measured by the percentage reaction inhibition rate of enzyme with WST-1 (Water Soluble Tetrazolium dye) substrate and xanthine oxidase using an SOD Assay Kit (Dojindo Laboratories, Kumamoto, Japan). The endpoint assay was read at an absorbance wavelength of 450 nm (the absorbance wavelength for the formazan dye product of WST-1 reaction with superoxide) after 20 min of reaction time at 37 °C. 

Blood chemistry parameters were analyzed by an automatic chemical analyzer (Fuji DRI-CHEM 3500i, Fuji Photo Film, Ltd., Tokyo, Japan), which was used to determine ALT, AST, TP, and GLU levels in serum.

A bacterial challenge test was performed at the end of the feeding trial by intraperitoneal injection with 1 × 10^8^ CFU *Streptococcus* *iniae* (*S. iniae*) obtained from the Department of Biotechnology (Pukyong National University, Busan, Rep. Korea) according to Hasan Md et al. [60]. Briefly, five fish from each tank per dietary treatment groups (n = 15) were fasted 24 h prior to being sedated by 2-phenoxyethanol, given an intraperitoneal injection with 100 μL of *S. iniae* (KCTC 3657) using sterile nonheparinized 1.0 mL syringes at a concentration of 1 × 10^8^ CFU/mL [61], divided into separate triplicate tanks in groups of five without recirculation, and supplied with airstones. The fish were not fed during the challenge test and mortality was recorded twice daily. This challenge test and all above-stated protocol were approved by the Animal Use and Care Committee of Pukyong National University (protocol number 554).

### 2.4. Statistical Analysis

All data were analyzed by one-way ANOVA to test for the effects of dietary treatment with GABA supplementation. When significant differences were found, Duncan’s Multiply Range Test (DMRT) was employed to evaluate differences among dietary treatment groups. Values were considered to be significant (i.e., the null hypothesis rejected) at a level of *p* ≤ 0.05. All statistical analyses were performed in SPSS 20 (IBM).

## 3. Results

### 3.1. Growth

**Figure 1:**
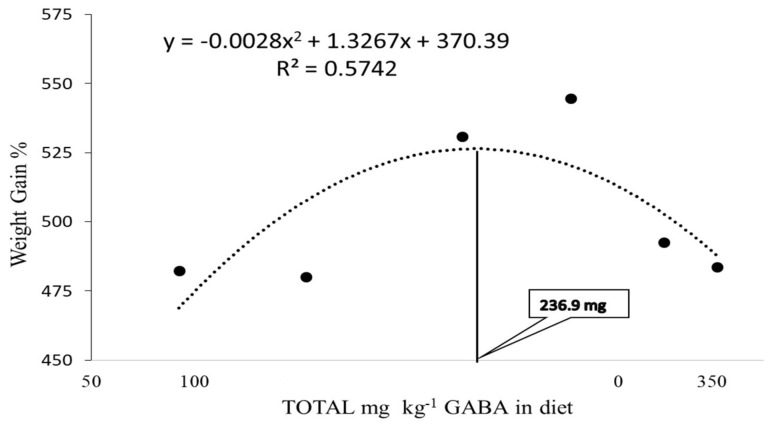
Optimal level of dietary GABA in mg/kg according to WG by polynomial analysis in Juvenile olive flounder fed the experimental diets (OTC was excluded in this analysis). GABA concentration on the X axis is according to actual GABA levels of diets: CON, GAB_50_, GAB_100_, GAB_150_, GAB_200_, and GAB_250_ as determined by HPLC analysis. See Table 1 for more information about formulation and GABA content.

At the end of the feeding trial, the average WG and SGR of fish fed GAB_100_ and GAB_150_ diets were significantly higher (*p* < 0.05) than those of fish fed CON, OTC, GAB_50_, and GAB_250_ diets (Table 2). Yet, there were no significant differences among fish fed GAB_100_ and GAB_200_ diets (*p* > 0.05). According to polynomial regression analysis (Figure 1) the optimal level (according to the total GABA content) for growth was estimated to be 236.9 mg/kg, which is a value that lies between the total GABA content of diets GAB_100_ and GAB_150_ (Table 1). There were no significant differences with regard to FE, VSI, HIS, survival, and final percent coverage area (fPCA) (Table 2).

### 3.2. Whole-Body Proximate Composition of Fish Fed Experimental Diets

Whole body composition of fish fed the experimental diets is presented in Table 3. There were no significant differences in whole-body moisture, crude protein, crude lipid and crude ash of fish fed all the experimental diets (*p* > 0.05). 

### 3.3. Digestive Enzyme

**Figure 2:**
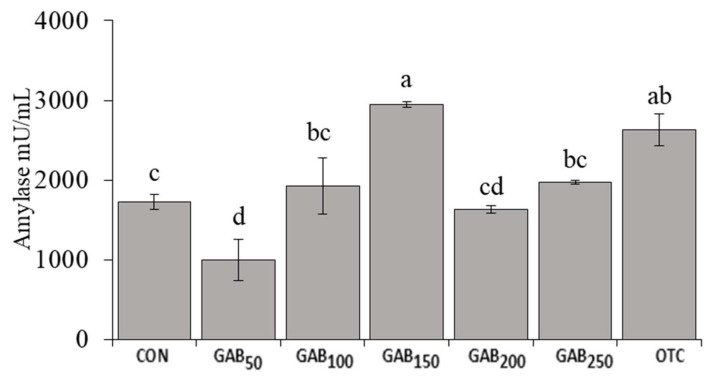
Intestinal amylase activity in juvenile olive flounder fed increasing levels of GABA. Values are mean of triplicate samples. Values with different letters are significantly different according to one-way ANOVA (*p* ≤ 0.05) and Duncan’s multiple range test (DMRT). See Table 1 for information on diets: CON, GAB50, GAB100, GAB150, GAB200, GAB250, and OTC.

**Figure 3:**
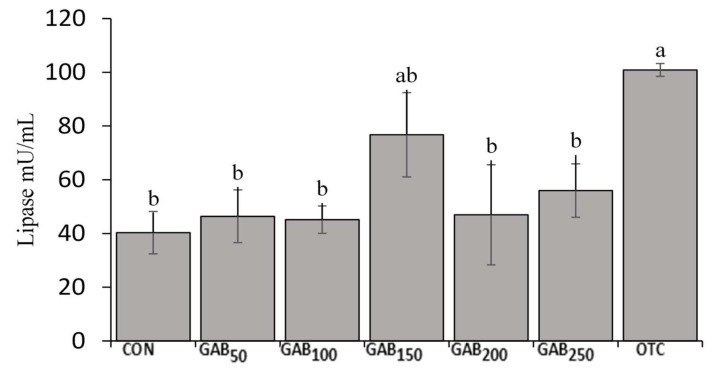
Intestinal lipase activity in juvenile olive flounder fed increasing levels of GABA. Values are mean of triplicate samples. Values with different letters are significantly different according to one-way ANOVA (*p* ≤ 0.05) and Duncan’s Multiple Range test (DMRT). See Table 1 for information on diets: CON, GAB_50_, GAB_100_, GAB_150_, GAB_200_, GAB_250_, and OTC.

As for intestinal digestive enzyme activity, only amylase activity presented a clear trend with significant results (Figure 2). The results show that fish fed OTC and GAB_150_ diets had significantly higher amylase activity than fish fed any other diet. With regard to lipase enzyme activity, there was a trend similar to that of amylase; however, the values were not distinct enough to be considered statistically significant in any diet except for the OTC diet (Figure 3).

### 3.4. Hematological Parameters

At the end of the feeding trial there were no significant differences in blood serum AST, ALT, GLU, or TP levels (Table 4).

### 3.5. Antioxidant and Non-Specific Immune Responses

**Figure 4:**
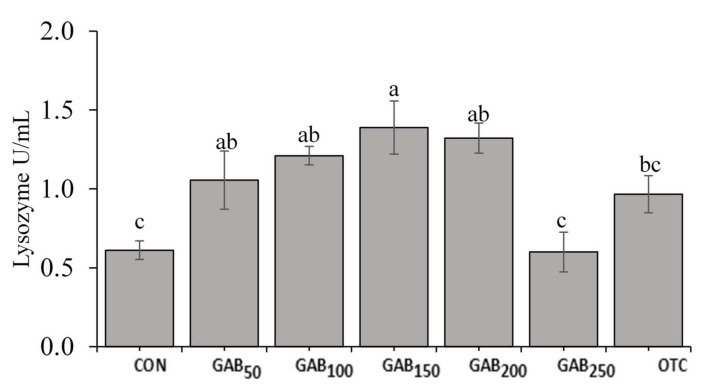
Serum lysozyme activity in juvenile olive flounder fed increasing levels of GABA. Values are mean of triplicate samples. Values with different letters are significantly different according to one-way ANOVA (*p* ≤ 0.05) and Duncan’s multiple range test (DMRT). See Table 1 for information on diets: CON, GAB_50_, GAB_100_, GAB_150_, GAB_200_, GAB_250_, and OTC.

**Figure 5:**
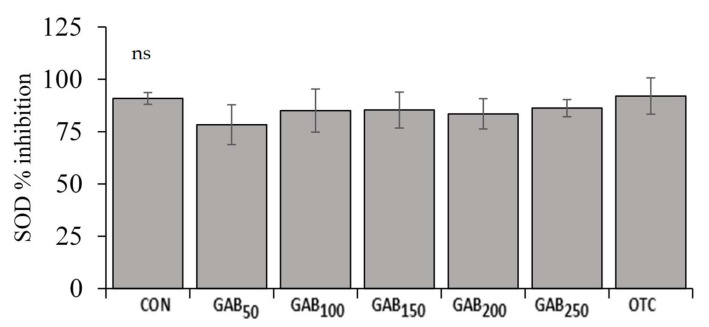
Serum superoxide dismutase (SOD) activity in juvenile olive flounder fed increasing levels of GABA. Values are mean of triplicate samples. See Table 1 for information on diets: CON, GAB_50_, GAB_100_, GAB_150_, GAB_200_, GAB_250_, and OTC.

**Figure 6:**
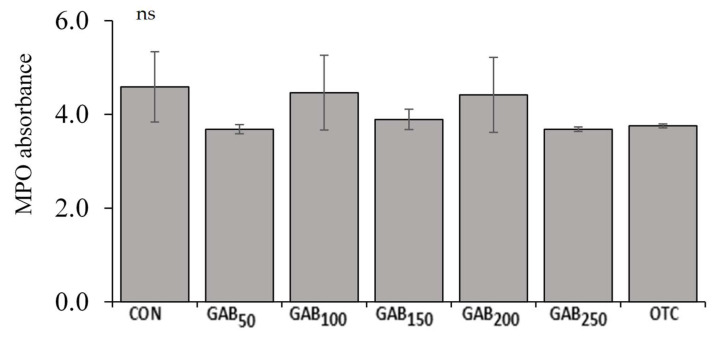
Serum myeloperoxidase (MPO) activity in juvenile olive flounder fed increasing levels of GABA. Values are mean of triplicate samples. See Table 1 for information on diets: CON, GAB_50_, GAB_100_, GAB_150_, GAB_200_, GAB_250_, and OTC.

The average lysozyme activities of fish fed GAB_50_, GAB_100_, GAB_150_, and GAB_200_ diets were significantly higher (*p* ≤ 0.05) than those of fish fed CON and GAB_250_ diets. However, there were neither significant differences among fish fed GAB_50_, GAB_100_, GAB_200_, and OTC diets, nor between GAB_250_ and OTC diets (Figure 4). There were no significant differences in SOD or MPO among fish fed any of the experimental diets (Figure 5 and Figure 6).

### 3.6. Challenge Test

**Figure 7:**
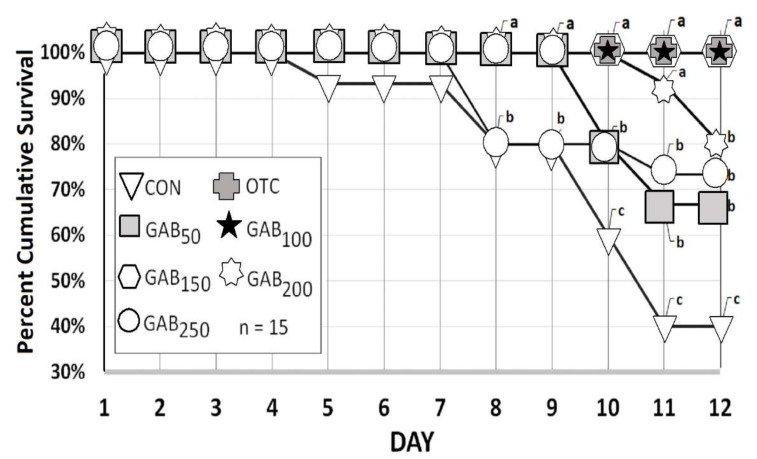
Percent cumulative survival of juvenile olive flounder fed increasing levels of GABA which were administered an intraperitoneal injection with 1 × 10^8^ CFU *Streptococcus* *iniae*. Values are the mean of triplicates of five fish grouped according to dietary treatment: n = 15. Values with different letters are significantly different according to one-way ANOVA (*p* ≤ 0.05) and Duncan’s multiple range test (DMRT) according to the day. See Table 1 for information on diets: CON, GAB_50_, GAB_100_, GAB_150_, GAB_200_, GAB_250_, and OTC.

The percent cumulative survival of all fish fed GABA-supplemented diets was significantly higher than that of fish fed a CON diet by the 10th day of the challenge test. Furthermore, by the 12th day, the percent survival of fish fed OTC, GAB_100_ and GAB_150_ diets was significantly higher than that of fish fed all the other diets (Figure 7).

### 3.7. Quantification of Optimum Supplementation of GABA for Olive Flounder

Taking significantly different parameters into account (i.e., WG, SGR, intestinal amylase, serum lysozyme, and cumulative survival of the challenge test), in terms of dietary treatment group, GAB_100_ and GAB_150_ consistently outperform other diets. This can further be supported by a polynomial regression analysis of significant growth parameters, which correlates well with other significant, non-growth-related parameters such as amylase activity and those related to immunity, such as disease resistance, as expressed in cumulative survival and lysozyme activity. Thus, in conjunction with ANOVA results, a quantification of optimum supplementation of GABA in this trial was assessed by utilizing a quadratic polynomial (second order) regression analysis employing the formula Y = aX^2^ + bX + C and applying it to growth according to Pesti et al. [62], in order to establish a value of approximately 237 mg/kg, which lies between diets GAB_100_ and GAB_150_ correlating to a total GABA content level of 229 and 282 mg/kg, respectively. 

## 4. Discussion

In this experiment, WG and SGR were significantly improved with the GAB_100_ and GAB_150_ diets, corresponding to a GABA content between 229 and 282 mg/kg. Similar results were found in experiments assessing an optimal level for GABA with grass carp [44] and tilapia [42]. One reason for the improvement in WG could come from the documented effects of GABA on the expression of the growth hormone [63]. The reason for impaired growth performance with an increased GABA supplementation level may require much deeper levels of analysis, such as the genetic expression of growth-related genes that may be affected when dietary GABA exceeds optimal levels. Though we do not have an indication of suppressed feeding behavior according to observation, and no statistically differences in FE in this trial, a following trial to precisely quantify feed intake is merited by utilizing a floating feed and lower stocking densities for a more precise estimation of feed intake. Nevertheless, earlier trials support an inhibition of feeding with high levels of GABA intake, as evidenced by Kim et al. [64]. These authors fed juvenile olive flounder with diets containing 1000 mg/kg of GABA and observed an inhibitory effect on feed intake. As mentioned before, fish in the current trial readily consumed all feed provided, and this is no doubt due to formulation with much lower levels of GABA in diets that are formulated to be nutritionally complete and highly palatable. To understand the mechanism of GABA’s effect on feed intake, a deeper level of analysis must be undertaken in future trials, as shown in the recent work by Zhang et al. [45], which found that dietary GABA supplementation in Chinese mitten crab (*Eriocheir sinensis*) is associated with an increased expression of orexigenic, neural, signal-related genes, while anorexigenic neural signal-related genes are decreased. Thus, the interplay between growth, orexigenic and anorexigenic genes, as well as the exploration of such complex factors as the gut microbiome, may provide a framework for understanding relationship between dietary GABA consumption and growth.

Digestive enzymes are an important class of biomolecule that breaks down nutrients from their complex polymeric structures into smaller, more digestible constituents. In this trial, we assessed the activity of amylase and lipase in the intestine of juvenile olive flounder. Our results showed that the amylase and lipase activities of fish fed OTC and GAB_150_ diets were significantly higher than those of fish fed all the other diets. To the best of our knowledge, this is the only trial assessing intestinal amylase and lipase activity in juvenile olive flounder. However, in similar trials, GABA was found to increase the activity of other digestive enzymes such as intestinal trypsin [43]. In laying hens, GABA was linked to the increased gastric enzyme activity of amylase and lipase [65]. Furthermore, there may be a link between GABA supplementation and intestinal enzyme activity in olive flounder by improvements in the gut-microbiota by serving as a pre/postbiotic supporting probiotic growth. In a trial by Ye et al. [66], diets supplemented with fructo and mannan oligosaccharide supplements and *Bacillus clausii* showed an enhancement in amylase activity. Probiotic *Bacillus* species are an important constituent of the gut microflora, which is a complex community of bacteria, viruses, and fungi that benefits from nutrients broken down during digestion, and in turn produces a variety of beneficial metabolites, such as GABA. As in all living things, this community of microorganisms produces and utilizes metabolites in their environment. An improvement in digestive enzyme activity from exogenous GABA may point to GABA’s utility as a modulator of the gut microbiome [67]. 

Non-specific immunity, also known as innate immunity, is the principal means of disease resistance in fish. Non-specific immune responses are nearly instantaneous, but lack the ability to retain the immunological memory that is associated with adaptive immunity. Non-specific immunity is the first line of defense and is believed to be more important than adaptive immunity, which is slower and less robust in fish, compared to higher-level vertebrates (e.g., mammals) [68,69]. An important enzyme involved in non-specific immune response, which has been widely studied in fish, is lysozyme. Lysozyme is an enzyme produced by leukocytes that hydrolyzes β-(1→4) glycosidic linkages between N-acetylmuramic acid and N-acetylglucosamine in the cell walls of Gram-positive bacteria [9]. In this trial, lysozyme activity was significantly improved in all GABA-supplemented diets as well as the OTC diet. GABA’s effects on lysozyme in this trial may be due to an increase in GABA-associated macrophage activation [70]. This increase in macrophage activation is then made evident by increased lysozyme activity [71]. Other important, non-specific immune responses are SOD and MPO. Though technically classified as antioxidant enzymes, SOD and MPO play an integral role in innate immunity due to their production in response to their elimination of reactive oxygen species (ROS) that are generated by immune cells, such as macrophages, in the process of destroying pathogens [72,73]. In this trial, neither SOD nor MPO were significantly different; however, there appeared to be a slight trend of upregulation. However, In Nile tilapia (*Oreochromis niloticus*), Temu et al. [42] found that fish fed a dietary level of 158 mg/kg had significantly increased SOD activity. This of course may be due to the many physiological, nutritional, and environmental differences between the species.

*Streptococcus iniae* is a bacterial pathogen of special concern to the aquaculture industry. *S. iniae* is a Gram-positive bacterium that has become a very serious threat to many aquaculture species: both freshwater and marine species [8]. Therefore, it was chosen to assess the disease resistance of the olive flounder in this experiment. As previously mentioned in the results, optimal GABA supplementation led to a noted improvement in cumulative survival. There were no significant differences between the two aforementioned diets compared to OTC, indicating that GABA could be used to reduce the use of OTC for olive flounder cultures, which is an unfortunate but common practice. This is important because pathogens wreaked havoc on the olive flounder industry in previous years. Production in the Republic of Korea peaked at 54,574 MT in 2009, but shortly thereafter fell into a sharp decline: to the level of 36,921 MT in 2014. This decrease in production was largely due to the outbreak of pathogens amongst olive flounder operations and caused a 32% decline. [2,7]. At any rate, a mechanism for pathogen resistance in the current trial may be gleaned from a recent paper regarding GABA’s effects on microbial defense, in which it was shown that GABA has antimicrobial effects via the modulation of macrophage activity by GABAAR-Ca^2+^- AMP-activated protein kinase signaling [70]. In this trial, GABAergic signaling was linked to autophage enhancement resulting in the infected host’s protection against intracellular bacterial infections. Treating macrophages with GABA or other GABAergic agents resulted in an increase in autophage activation, leading to phagosomal maturation, and thus an improved antimicrobial response. Additionally, research was conducted recently on the gut microbiome, in which Strandwitz et al. [28] found that GABA was essential for the growth of a newly discovered bacteria (KLE1738). To our knowledge this was the first trial that assessed GABA’s effects on the survival of a bacterial challenge in marine flatfish. Additionally, juvenile olive flounder were used in this study, and there are likely to be different results with adult or broodstock olive flounder. This is because GABA is known to have different effects during very early development than in later stages, especially when considering the stages of embryonic development [74,75]. A trial accessing GABA’s effects at different live stages ranging from larval to brood stock with analyses such as flow cytometry in conjunction with histology [76,77,78,79,80], in order to evaluate immune-related effects more precisely, is warranted.

## 5. Conclusions

In this trial the optimal level of dietary GABA (total endogenous and supplemented) was calculated to be approximately 237 mg/kg, according to the polynomial regression model based on WG (Figure 1). Results from the digestive enzyme, lysozyme activity, and the *S. iniae* challenge test aligned with the results of WG (Figure 7), which closely corresponded to the supplementation levels of diets GAB_100_ and GAB_150_ or 229 and 282 mg/kg, respectively. Given the affordability of GABA, it is a relatively small inclusion proportional to other ingredients and their efficacy. For this reason, GABA is likely to gain continued attention, especially as a feed additive. Lastly, since GABA is also an important metabolite of bacterial metabolism, future trials should be designed to assess the ability of GABA to modulate the gut microbiome and genetic expression of key developmental/growth-related biomarkers via emerging multi-omic technologies.

## Figures and Tables

**Table 1:** Formulation of the seven experimental diets ^†^ supplemented with different levels of γ-aminobutyric acid (GABA) and fed to olive flounder for eight weeks (g/kg dry matter (DM) bases).

Ingredients	CON	GAB_50_	GAB_100_	GAB_150_	GAB_200_	GAB_250_	OTC
Sardine FM ^a^	250	250	250	250	250	250	250
Anchovy FM ^a^	250	250	250	250	250	250	250
Soybean meal ^b^	150	150	150	150	150	150	150
Wheat flour ^b^	130	129.93	129.87	129.80	129.74	129.67	126
Squid liver powder ^a^	40	40	40	40	40	40	40
Meat and bone meal	50	50	50	50	50	50	50
Poultry BP	40	40	40	40	40	40	40
Fish oil	42	42	42	42	42	42	42
Lecithin	5	5	5	5	5	5	5
Betaine	10	10	10	10	10	10	10
Taurine	5	5	5	5	5	5	5
MCP ^c^	5	5	5	5	5	5	5
Mineral mix ^d^	10	10	10	10	10	10	10
Vitamin mix ^e^	10	10	10	10	10	10	10
Choline	3	3	3	3	3	3	3
Oxytetracycline	--	--	--	--	--	--	4
GABA (76.5% purity) ^f^	0	0.065	0.131	0.196	0.261	0.327	0
Total	1000	1000	1000	1000	1000	1000	1000
Total GABA content in mg/kg according to HPLC ^1,2^							
Total GABA	92.36	153.87	229.27	281.79	326.68	352.28	102.99
Proximate composition of experimental diets (DM) % ^2,3^							
Moisture	8.22 ± 0.04	8.18 ± 0.01	8.73 ± 0.10	8.74 ± 0.05	8.60 ± 0.05	9.65 ± 0.07	8.74 ± 0.01
Crude protein	51.9 ± 0.44	52.1 ± 0.07	52.2 ± 0.07	51.8 ± 0.13	52.0 ± 0.31	51.6 ± 0.00	52.0 ± 0.07
Crude lipid	9.92 ± 0.25	10.14 ± 0.20	10.21 ± 0.18	9.88 ± 0.02	10.39 ± 0.15	9.93 ± 0.09	9.40 ± 0.09
Ash	12.0 ± 0.06	12.4 ± 0.29	12.1 ± 0.11	12.3 ± 0.27	12.5 ± 0.18	12.0 ± 0.22	12.10 ± 0.22

^†^ Diet names reflect the level of supplemental GABA added to the diet. ^a^ Suhyup feed Co. Uiryeong, Korea. ^b^ The feed Co. Goyang, Korea. ^c^ MCP (CaHPO_4_). Sigma-Aldrich Korea Yongin, Korea. ^d^ Contains (as mg/kg in diets): Ascorbic acid, 300; dl-Calcium panthothenate, 150; Choline bitatrate, 3000; Inositol, 150; Menadione, 6; Niacin, 150; Pyridoxine·HCl, 15; Riboflavin, 30; Thiamine mononitrate, 15; dl-α-tocopherol acetate, 201; Retinyl acetate, 6; Biotin, 1.5; Folic acid, 5.4; B_12_, 0.06. ^e^ Contains (as mg/kg in diets): NaCl, 437; MgSO_4_·7H_2_O, 1380; NaH_2_P_4_·2H_2_O, 878; Ca(H_2_PO_4_)·2H_2_O, 1367; KH_2_PO_4_, 2414; ZnSO_4_·7H_2_O, 226; Fe-Citrate, 299; Ca-lactate, 3004; MnSO_4_, 0.016; FeSO_4_, 0.0378; CuSO_4_, 0.00033; Calcium iodate, 0.0006; MgO, 0.00135; NaSeO_3_, 0.00025. ^f^ Milae Bioresources Co., Seoul, Rep Korea ^1^ HPLC was performed at the National Instrumentation Center for Environmental Management College of Agriculture and Life Sciences at Seoul National University (Seoul 151–742, Korea) ^2^ Values are mean of duplicate samples. ^3^ Values with different letters within the same row are significantly different according to one-way ANOVA (*p* ≤ 0.05) and Duncan’s multiple range test (DMRT) while ‘ns’ means no significant difference.

**Table 2:** Growth performance, feed efficiency, organosomatic indices, survival, and fPCA ^1^.

Diets ^2^									
	CON	GAB_50_	GAB_100_	GAB_150_	GAB_200_	GAB_250_	OTC	R^2^	*p*-Value
IBW ^3^	4.86 ± 0.05	4.96 ± 0.03	4.94 ± 0.07	4.97 ± 0.05	4.90 ± 0.07	4.91 ± 0.06	4.81 ± 0.03	-	0.3989
WG ^4^	482 ± 14.90 ^c^	480 ± 20.92 ^c^	531 ± 16.34 ^ab^	545 ± 15.99 ^a^	493 ± 7.89 ^bc^	484 ± 3.31 ^c^	475 ± 7.50 ^c^	0.574	0.0143
SGR ^5^	3.04 ± 0.04 ^c^	3.03 ± 0.06 ^c^	3.17 ± 0.05 ^ab^	3.21 ± 0.04 ^a^	3.07 ± 0.02 ^bc^	3.04 ± 0.01 ^c^	3.02 ± 0.02 ^c^	0.561	0.0156
FE ^6^	121 ± 4.00	119 ± 7.89	130 ± 3.92	128 ± 4.70	113 ± 2.55	114 ± 0.08	116 ± 1.78	-	0.1018
VSI ^7^	1.50 ± 0.08	1.48 ± 0.04	1.45 ± 0.07	1.44 ± 0.06	1.50 ± 0.04	1.56 ± 0.07	1.67 ± 0.14	-	0.4119
HSI ^8^	0.87 ± 0.11	0.73 ± 0.04	0.65 ± 0.02	0.72 ± 0.04	0.75 ± 0.04	0.86 ± 0.08	0.79 ± 0.08	-	0.2337
Survival (%) ^9^	97.8 ± 1.11	96.7 ± 3.33	93.3 ± 1.92	90.0 ± 3.33	88.9 ± 1.11	90 ± 3.33	94.4 ± 2.22	-	0.1465
^†^ fPCA (%) ^10^	103 ± 2.12	99.9 ± 5.32	106 ± 1.15	101 ± 5.56	93.5 ± 3.00	95.7 ± 1.71	97.9 ± 0.92	-	0.2107

^1^ Values are mean of triplicate samples. Values with different letters within the same row are significantly different according to one-way ANOVA (*p* ≤ 0.05) and Duncan’s multiple range test (DMRT). ^2^ See Table 1 for information on experimental diets. ^3^ Initial body weight (g). ^4^ Weight gain (%) = (final wt. − initial wt.) × 100/initial wt. ^5^ Specific growth rate (%) = (ln final weight − ln initial weight) × 100/d. ^6^ Feed efficiency (%) = wet weight gain × 100/dry feed intake. ^7^ Viscerosomatic index (VSI) = 100 × viscera weight (g)/body weight (g) ^8^ Hepatosomatic index (%) = liver weight × 100/body weight. ^9^ Percent survival (%) = (survival fish − dead fish) × 100/survival fish ^10^ Final percent coverage area (%) = total ventral surface of fish in cm^2^/total tanks bottom area × 100. ^†^ initial PCA was 38% for all tanks. R^2^ values in this table derive from quadratic (polynomial) regression analysis. For calculation of R^2^ value, OTC was excluded.

**Table 3:** Proximate composition (DM) % of juvenile olive flounder fed experimental diets ^1^.

Diet ^2^								
	CON	GAB_50_	GAB_100_	GAB_150_	GAB_200_	GAB_250_	OTC	*p*-Value
Crude Protein	71.43 ± 0.67	71.48 ± 0.89	70.26 ± 0.32	70.52 ± 1.11	71.55 ± 0.70	71.59 ± 1.10	71.34 ± 0.45	0.8276
Crude Lipid	11.53 ± 0.58	12.07 ± 0.42	10.90 ± 1.33	12.17 ± 0.20	12.12 ± 0.49	11.58 ± 1.05	10.87 ± 0.77	0.7923
Crude Ash	15.51 ± 0.22	16.81 ± 0.39	16.33 ± 0.40	15.92 ± 0.55	15.37 ± 0.14	16.25 ± 0.71	15.94 ± 0.95	0.5697
Moisture	74.51 ± 0.73	74.33 ± 0.36	75.89 ± 0.51	74.61 ± 0.45	75.07 ± 0.17	75.91 ± 0.17	75.96 ± 0.44	0.0638

^1^ Values are mean of triplicate samples, ^2^ See Table 1 for information on experimental diets.

**Table 4:** Blood chemistry of juvenile olive flounder ^1.^

Diet ^2^								
	CON	GAB_50_	GAB_100_	GAB_150_	GAB_200_	GAB_250_	OTC	*p*-Value
AST U/L ^3^	16.0 ± 0.58	13.3 ± 1.45	15.3 ± 2.96	15.3 ± 1.67	17.7 ± 3.71	16.7 ± 2.67	15.7 ± 2.19	0.9177
ALT U/L ^4^	5.00 ± 0.00	4.33 ± 0.33	5.00 ± 0.58	5.00 ± 0.00	4.33 ± 0.33	6.00 ± 0.58	4.33 ± 0.67	0.1481
GLU mg/dL ^5^	17.3 ± 1.76	22.0 ± 4.51	22.0 ± 3.51	24.3 ± 4.18	23.3 ± 2.73	19.7 ± 5.17	29.0 ± 11.24	0.8368
TP g/dL ^6^	3.27 ± 0.18	3.03 ± 0.29	3.43 ± 0.39	3.87 ± 0.41	4.23 ± 0.85	3.83 ± 0.73	3.97 ± 0.66	0.7254

^1^ Values are mean of triplicate samples (Fuji DRI-CHEM 3500i, Fuji Photo Film, Ltd., Tokyo, Japan). ^2^ See Table 1 for more information on experimental diets. ^3^ AST (U/L): Aspartate aminotransferase. ^4^ ALT (U/L): Alanine aminotransferase. ^5^ GLU (mg/dL): Serum glucose. ^6^ TP (g/dL): Serum total protein.

## Data Availability

The data that support the findings of this study are available on request from the corresponding author. The data are not publicly available due to privacy or ethical restrictions.

## References

[1] Hamidoghli A., Won S., Lee S., Lee S., Farris N.W., Bai S.C. (2020). Nutrition and feeding of olive flounder *Paralichthys olivaceus*: A Review. Rev. Fish. Sci. Aquac..

[2] FAO Global Production Online Query. http://www.fao.org/fishery/statistics/global-production/en.

[3] Katya K., Park G., Bharadwaj A.S., Browdy C.L., Vazquez-Anon M., Bai S.C. (2018). Organic acids blend as dietary antibiotic replacer in marine fish olive flounder, *Paralichthys olivaceus*. Aquac. Res..

[4] Park Y., Park M., Hamidoghli A., Kim C.-H., Bai S.C. (2021). Optimum dietary processed sulfur (Immuno-F) level has antibiotic effects on the growth, hematology and disease resistance of juvenile olive flounder, *Paralichthys olivaceus*. Anim. Feed Sci. Technol..

[5] Lee Y.K., Katya K., Yun H.H., Yoon M.Y., Park J.K., Sung J.S., Shin H.S., Bai S.C. (2016). Evaluation of dietary yellow loess as an antibiotic replacer on growth, immune responses, serological characteristics and disease resistance in rainbow trout, Oncorhynchus mykiss. Aquac. Nutr..

[6] Bai S.C., Katya K., Yun H. (2015). Additives in aquafeed: An overview. Feed Feed. Pract. Aquac..

[7] Hasan M.T., Je Jang W., Lee J.M., Lee B.-J., Hur S.W., Gu Lim S., Kim K.W., Han H.-S., Kong I.-S. (2019). Effects of immunostimulants, prebiotics, probiotics, synbiotics, and potentially immunoreactive feed additives on olive flounder (*Paralichthys olivaceus*): A review. Rev. Fish. Sci. Aquac..

[8] Park Y.-K., Nho S.-W., Shin G.-W., Park S.-B., Jang H.-B., Cha I.-S., Ha M.-A., Kim Y.-R., Dalvi R.S., Kang B.-J. (2009). Antibiotic susceptibility and resistance of Streptococcus iniae and Streptococcus parauberis isolated from olive flounder (*Paralichthys olivaceus*). Vet. Microbiol..

[9] Gatlin D.M., Yamamoto F.Y. (2021). Nutritional supplements and fish health. Fish Nutrition.

[10] Storici P., De Biase D., Bossa F., Bruno S., Mozzarelli A., Peneff C., Silverman R.B., Schirmer T. (2004). Structures of γ-aminobutyric acid (GABA) aminotransferase, a pyridoxal 5′-phosphate, and [2Fe-2S] cluster-containing enzyme, complexed with γ-ethynyl-GABA and with the antiepilepsy drug vigabatrin. J. Biol. Chem..

[11] Khan W., Bhatt P.C., Panda B.P. (2015). Degradation Kinetics of Gamma Amino Butyric Acid in M onascus-Fermented Rice. J. Food Qual..

[12] Serrano-Regal M.P., Bayón-Cordero L., Ordaz R.P., Garay E., Limon A., Arellano R.O., Matute C., Sánchez-Gómez M.V. (2020). Expression and function of GABA receptors in myelinating cells. Front. Cell. Neurosci..

[13] Hepsomali P., Groeger J.A., Nishihira J., Scholey A. (2020). Effects of oral gamma-aminobutyric acid (GABA) administration on stress and sleep in humans: A systematic review. Front. Neurosci..

[14] Harilal S., Kumar R., Mathew G.E., Jose J., Uddin M.S., Mathew B. (2020). Neurochemicals in nervous system and exploring the chemical make-up of human brain. Principles of Neurochemistry.

[15] Bhagat K., Singh J.V., Pagare P.P., Kumar N., Sharma A., Kaur G., Kinarivala N., Gandu S., Singh H., Sharma S. (2021). Rational approaches for the design of various GABA modulators and their clinical progression. Mol. Divers..

[16] Vlachou S. (2021). GABA B Receptors and Cognitive Processing in Health and Disease. Current Topics in Behavioral Neurosciences.

[17] Brohan J., Goudra B.G. (2017). The role of GABA receptor agonists in anesthesia and sedation. CNS Drugs.

[18] Hulme A.C., Arthington W. (1950). γ-Amino-butyric acid and β-alanine in plant tissues: Amino-acids of the apple fruit. Nature.

[19] Awapara J., Landua A.J., Fuerst R., Seale B. (1950). Free γ-aminobutyric acid in brain. J. Biol. Chem..

[20] Fenalti G., Law R.H.P., Buckle A.M., Langendorf C., Tuck K., Rosado C.J., Faux N.G., Mahmood K., Hampe C.S., Banga J.P. (2007). GABA production by glutamic acid decarboxylase is regulated by a dynamic catalytic loop. Nat. Struct. Mol. Biol..

[21] Graham L.T., Baxter C.F., Lolley R.N. (1970). In vivo influence of light or darkness on the GABA system in the retina of the frog (*Rana pipiens*). Brain Res..

[22] Fugelli K. (1970). Gamma-aminobutyric acid (GABA) in fish erythrocytes. Experientia.

[23] Morse D.E., Hooker N., Duncan H., Jensen L. (1979). γ-Aminobutyric acid, a neurotransmitter, induces planktonic abalone larvae to settle and begin metamorphosis. Science.

[24] Wilkinson M., Wilkinson D.A., Khan I., Crim L.W. (1983). Benzodiazepine receptors in fish brain: [3H]-flunitrazepam binding and modulatory effects of GABA in rainbow trout. Brain Res. Bull..

[25] Roseth S., Fonnum F. (1995). A study of the uptake of glutamate, γ-aminobutyric acid (GABA), glycine and β-alanine in synaptic brain vesicles from fish and avians. Neurosci. Lett..

[26] Kinnersley A.M., Turano F.J. (2000). Gamma aminobutyric acid (GABA) and plant responses to stress. CRC. Crit. Rev. Plant Sci..

[27] Watanabe M., Maemura K., Kanbara K., Tamayama T., Hayasaki H. (2002). GABA and GABA receptors in the central nervous system and other organs. Int. Rev. Cytol..

[28] Strandwitz P., Kim K.H., Terekhova D., Liu J.K., Sharma A., Levering J., McDonald D., Dietrich D., Ramadhar T.R., Lekbua A. (2019). GABA-modulating bacteria of the human gut microbiota. Nat. Microbiol..

[29] McCormick D.A. (1989). GABA as an inhibitory neurotransmitter in human cerebral cortex. J. Neurophysiol..

[30] Schousboe A., Bak L.K., Waagepetersen H.S. (2013). Astrocytic control of biosynthesis and turnover of the neurotransmitters glutamate and GABA. Front. Endocrinol..

[31] Bown A.W., Shelp B.J. (2020). Does the GABA shunt regulate cytosolic GABA?. Trends Plant Sci..

[32] Cavalcanti-de-Albuquerque J.P., de-Souza-Ferreira E., de Carvalho D.P., Galina A. (2021). Coupling of GABA Metabolism to Mitochondrial Glucose Phosphorylation. Neurochem. Res..

[33] Liao J., Cao Y., Ren T., Shen Q., Wang Y., Ma Y., Fang W., Zhu X. (2020). GABA shunt contribution to flavonoid biosynthesis and metabolism in tea plants (*Camellia sinensis*); *Plant Physiol*. Biochem..

[34] Walls A.B., Waagepetersen H.S., Bak L.K., Schousboe A., Sonnewald U. (2015). The glutamine-glutamate/GABA cycle: Function, regional differences in glutamate and GABA production and effects of interference with GABA metabolism. Neurochem. Res..

[35] Andersen J.V., Markussen K.H., Jakobsen E., Schousboe A., Waagepetersen H.S., Rosenberg P.A., Aldana B.I. (2021). Glutamate metabolism and recycling at the excitatory synapse in health and neurodegeneration. Neuropharmacology.

[36] Abdou A.M., Higashiguchi S., Horie K., Kim M., Hatta H., Yokogoshi H. (2006). Relaxation and immunity enhancement effects of γ-aminobutyric acid (GABA) administration in humans. Biofactors.

[37] Shizuka F., Kido Y., Nakazawa T., Kitajima H., Aizawa C., Kayamura H., Ichijo N. (2004). Antihypertensive effect of γ-amino butyric acid enriched soy products in spontaneously hypertensive rats. Biofactors.

[38] Dhakal R., Bajpai V.K., Baek K.-H. (2012). Production of GABA (γ-aminobutyric acid) by microorganisms: A review. Braz. J. Microbiol..

[39] Mika A., Fleshner M. (2016). Early-life exercise may promote lasting brain and metabolic health through gut bacterial metabolites. Immunol. Cell Biol..

[40] Strandwitz P. (2018). Neurotransmitter modulation by the gut microbiota. Brain Res..

[41] Yang-Ho C. (2019). Effects of γ-aminobutyric acid on mortality in laying hens during summer time. J. Agric. Life Environ. Sci..

[42] Temu V., Kim H., Hamidoghli A., Park M., Won S., Oh M., Han J.-K., Bai S.C. (2019). Effects of dietary gamma-aminobutyric acid in juvenile Nile tilapia, *Orechromis niloticus*. Aquaculture.

[43] Bae J., Hamidoghli A., Won S., Choi W., Lim S.-G., Kim K.-W., Lee B.-J., Hur S.-W., Bai S.C. (2020). Evaluation of seven different functional feed additives in a low fish meal diet for olive flounder, *Paralichthys olivaceus*. Aquaculture.

[44] Wu F., Liu M., Chen C., Chen J., Tan Q. (2016). Effects of dietary gamma aminobutyric acid on growth performance, antioxidant status, and feeding-related gene expression of juvenile grass carp, *Ctenopharyngodon idellus*. J. World Aquac. Soc..

[45] Zhang C., Wang X., Su R., He J., Liu S., Huang Q., Qin C., Zhang M., Qin J., Chen L. (2022). Dietary gamma-aminobutyric acid (GABA) supplementation increases food intake, influences the expression of feeding-related genes and improves digestion and growth of Chinese mitten crab (*Eriocheir sinensis*). Aquaculture.

[46] Li C., Tian Y., Ma Q., Zhang B. (2022). Dietary gamma-aminobutyric acid ameliorates growth impairment and intestinal dysfunction in turbot (*Scophthalmus maximus* L.) fed a high soybean meal diet. Food Funct..

[47] Carabotti M., Scirocco A., Maselli M.A., Severi C. (2015). The gut-brain axis: Interactions between enteric microbiota, central and enteric nervous systems. Ann. Gastroenterol. Q. Publ. Hell. Soc. Gastroenterol..

[48] Hill J.M., Bhattacharjee S., Pogue A.I., Lukiw W.J. (2014). The gastrointestinal tract microbiome and potential link to Alzheimer’s disease. Front. Neurol..

[49] Chand N., Muhammad S., Khan R.U., Alhidary I.A., ur Rehman Z. (2016). Ameliorative effect of synthetic γ-aminobutyric acid (GABA) on performance traits, antioxidant status and immune response in broiler exposed to cyclic heat stress. Environ. Sci. Pollut. Res..

[50] Li Y.H., Li F., Liu M., Yin J.J., Cheng B.J., Shi B.M., Shan A.S. (2015). Effect of γ-aminobutyric acid on growth performance, behavior and plasma hormones in weaned pigs. Can. J. Anim. Sci..

[51] Mamuad L.L., Lee S.-S. (2021). The Role of Glutamic Acid-producing Microorganisms in Rumen Microbial Ecosystems. J. Life Sci..

[52] Ncho C.M., Jeong C., Gupta V., Goel A. (2021). The effect of gamma-aminobutyric acid supplementation on growth performances, immune responses, and blood parameters of chickens reared under stressful environment: A meta-analysis. Environ. Sci. Pollut. Res..

[53] Bai S.C., Lee S. (2010). Culture of olive flounder: Korean perspective. Practical Flatfish Culture and Stock Enhancement.

[54] CA AOAC (2005). Official methods of analysis of the Association of Analytical Chemists International.

[55] Iwata N., Kikuchi K., Honda H., Kiyono M., Kurokura H. (1994). Effects of temperature on the growth of Japanese flounder. Fish. Sci..

[56] National Institute of Fisheries Science. https://www.nifs.go.kr/fishfeed/view/supply/Calc.jsp.

[57] Charles Bai S., Hardy R.W., Hamidoghli A. (2022). Diet analysis and evaluation. Fish Nutr..

[58] Hultmark D., Steiner H., Rasmuson T., Boman H.G. (1980). Insect immunity. Purification and properties of three inducible bactericidal proteins from hemolymph of immunized pupae of *Hyalophora cecropia*. Eur. J. Biochem..

[59] Quade M.J., Roth J.A. (1997). A rapid, direct assay to measure degranulation of bovine neutrophil primary granules. Vet. Immunol. Immunopathol..

[60] Hasan M.T., Jang W.J., Kim H., Lee B.-J., Kim K.W., Hur S.W., Lim S.G., Bai S.C., Kong I.-S. (2018). Synergistic effects of dietary Bacillus sp. SJ-10 plus β-glucooligosaccharides as a synbiotic on growth performance, innate immunity and streptococcosis resistance in olive flounder (*Paralichthys olivaceus*). Fish Shellfish Immunol..

[61] Heo W.-S., Kim Y.-R., Kim E.-Y., Bai S.C., Kong I.-S. (2013). Effects of dietary probiotic, Lactococcus lactis subsp. lactis I2, supplementation on the growth and immune response of olive flounder (*Paralichthys olivaceus*). Aquaculture.

[62] Pesti G.M., Vedenov D., Cason J.A., Billard L. (2009). A comparison of methods to estimate nutritional requirements from experimental data. Br. Poult. Sci..

[63] Powers M.E., Yarrow J.F., McCoy S.C., Borst S.E. (2008). Growth hormone isoform responses to GABA ingestion at rest and after exercise. Med. Sci. Sports Exerc..

[64] Kim S.-K., Takeuchi T., Yokoyama M., Murata Y. (2003). Effect of dietary supplementation with taurine, β-alanine andGABA on the growth of juvenile and fingerling Japanese flounder *Paralichthys olivaceus*. Fish. Sci..

[65] Zhang M., ZOU X., Li H., DONG X., Zhao W. (2012). Effect of dietary γ-aminobutyric acid on laying performance, egg quality, immune activity and endocrine hormone in heat-stressed Roman hens. Anim. Sci. J..

[66] Ye J.-D., Wang K., Li F.-D., Sun Y.-Z. (2011). Single or combined effects of fructo-and mannan oligosaccharide supplements and Bacillus clausii on the growth, feed utilization, body composition, digestive enzyme activity, innate immune response and lipid metabolism of the Japanese flounder Paralichthy. Aquac. Nutr..

[67] Diez-Gutiérrez L., San Vicente L., Barron L.J.R., del Carmen Villaran M., Chávarri M. (2020). Gamma-aminobutyric acid and probiotics: Multiple health benefits and their future in the global functional food and nutraceuticals market. J. Funct. Foods..

[68] Magnadóttir B. (2006). Innate immunity of fish (overview). Fish Shellfish Immunol..

[69] Smith N.C., Rise M.L., Christian S.L. (2019). A comparison of the innate and adaptive immune systems in cartilaginous fish, ray-finned fish, and lobe-finned fish. Front. Immunol..

[70] Kim J.K., Kim Y.S., Lee H.-M., Jin H.S., Neupane C., Kim S., Lee S.-H., Min J.-J., Sasai M., Jeong J.-H. (2018). GABAergic signaling linked to autophagy enhances host protection against intracellular bacterial infections. Nat. Commun..

[71] Keshav S., Chung P., Milon G., Gordon S. (1991). Lysozyme is an inducible marker of macrophage activation in murine tissues as demonstrated by in situ hybridization. J. Exp. Med..

[72] Marikovsky M., Ziv V., Nevo N., Harris-Cerruti C., Mahler O. (2003). Cu/Zn superoxide dismutase plays important role in immune response. J. Immunol..

[73] Wang L., Wu Z.-Q., Wang X.-L., Ren Q., Zhang G.-S., Liang F.-F., Yin S.-W. (2016). Immune responses of two superoxide dismutases (SODs) after lipopolysaccharide or Aeromonas hydrophila challenge in pufferfish, Takifugu obscurus. Aquaculture.

[74] Hsu Y.-T., Chang Y.-G., Chern Y. (2018). Insights into GABAAergic system alteration in Huntington’s disease. R. Soc. Open Biol..

[75] Ncho C.-M., Goel A., Jeong C.-M., Youssouf M., Choi Y.-H. (2021). In Ovo Injection of GABA Can Help Body Weight Gain at Hatch, Increase Chick Weight to Egg Weight Ratio, and Improve Broiler Heat Resistance. Animals.

[76] Kiron V., Park Y., Siriyappagouder P., Dahle D., Vasanth G.K., Dias J., Fernandes J.M.O., Sørensen M., Trichet V.V. (2020). Intestinal transcriptome analysis reveals soy derivative-linked changes in Atlantic salmon. Front. Immunol..

[77] Fazio F., Saoca C., Costa G., Zumbo A., Piccione G., Parrino V. (2019). Flow cytometry and automatic blood cell analysis in striped bass Morone saxatilis (Walbaum, 1792): A new hematological approach. Aquaculture.

[78] Park Y., Abihssira-García I.S., Thalmann S., Wiegertjes G.F., Barreda D.R., Olsvik P.A., Kiron V. (2020). Imaging flow cytometry protocols for examining phagocytosis of microplastics and bioparticles by immune cells of aquatic animals. Front. Immunol..

[79] Acar Ü., Kesbiç O.S., Yılmaz S., İnanan B.E., Zemheri-Navruz F., Terzi F., Fazio F., Parrino V. (2021). Effects of Essential Oil Derived from the Bitter Orange (*Citrus aurantium*) on Growth Performance, Histology and Gene Expression Levels in Common Carp Juveniles (*Cyprinus carpio*). Animals.

[80] Parrino V., Cappello T., Costa G., Cannavà C., Sanfilippo M., Fazio F., Fasulo S. (2018). Comparative study of haematology of two teleost fish (*Mugil cephalus* and *Carassius auratus*) from different environments and feeding habits. Eur. Zool. J..

